# Disease-Homologous Mutation in the Cation Diffusion Facilitator Protein MamM Causes Single-Domain Structural Loss and Signifies Its Importance

**DOI:** 10.1038/srep31933

**Published:** 2016-08-23

**Authors:** Shiran Barber-Zucker, René Uebe, Geula Davidov, Yotam Navon, Dror Sherf, Jordan H. Chill, Itamar Kass, Ronit Bitton, Dirk Schüler, Raz Zarivach

**Affiliations:** 1 Department of Life Sciences and The National Institute for Biotechnology in the Negev, Ben-Gurion University of the Negev, Beer Sheva, 8410501, Israel; 2 Department of Microbiology, University of Bayreuth, Bayreuth, 95447, Germany; 3 Department of Chemical Engineering and Ilse Katz Institute for Nanoscale Science and Technology, Ben-Gurion University of the Negev, Beer Sheva, 8410501, Israel; 4 Department of Chemistry, Bar-Ilan University, Ramat-Gan, 5290002, Israel

## Abstract

Cation diffusion facilitators (CDF) are highly conserved, metal ion efflux transporters that maintain divalent transition metal cation homeostasis. Most CDF proteins contain two domains, the cation transporting transmembrane domain and the regulatory cytoplasmic C-terminal domain (CTD). MamM is a magnetosome-associated CDF protein essential for the biomineralization of magnetic iron-oxide particles in magnetotactic bacteria. To investigate the structure-function relationship of CDF cytoplasmic domains, we characterized a MamM M250P mutation that is synonymous with the disease-related mutation L349P of the human CDF protein ZnT-10. Our results show that the M250P exchange in MamM causes severe structural changes in its CTD resulting in abnormal reduced function. Our *in vivo*, *in vitro* and *in silico* studies indicate that the CTD fold is critical for CDF proteins’ proper function and support the previously suggested role of the CDF cytoplasmic domain as a CDF regulatory element. Based on our results, we also suggest a mechanism for the effects of the ZnT-10 L349P mutation in human.

Divalent transition metal cations, such as Zn^2+^, Cu^2+^, Ni^2+^, Mn^2+^ and Fe^2+^, are essential for many cellular functions such as DNA synthesis, transcription, enzyme activity, intracellular signaling or apoptosis^1^,^2^. Despite their importance, accumulation of these cations in cells can be cytotoxic^2^. Therefore, maintenance of metal cation homeostasis is crucial for cell function^2^. The cation diffusion facilitator (CDF) protein family, a conserved family of transmembrane transporters found in all domains of life, ensures divalent transition metal cation homeostasis at the cellular level^3^. Most family members are antiporters, catalyzing the transport of divalent transition metal cations from the cytoplasm to the extracellular matrix or into intracellular compartments by exploiting the proton motive force^4^. CDF proteins usually form homodimers and share a conserved structure containing a transmembrane domain (TMD) with six transmembrane α-helices and a cytoplasmic N-terminus. Most CDF family members also have a relatively large cytoplasmic C-terminal domain (CTD), which adopts a metallochaperone-like fold and has been suggested to have a regulatory role in the transport mechanism^5^,^6^,^7^,^8^,^9^,^10^,^11^. Upon metal binding the CTDs change their conformation, resulting in a more compact fold and tighter association of two CTDs from different protomers, which in turn induces a conformational change of the TMD that allows metal/proton (Me^2+^/H^+^) antiport by an alternating-access mechanism^5^.

The human Zinc Transporter proteins (ZnT-1-10, also known as SLC30A1-10) are CDF proteins involved in zinc or manganese homeostasis and are critical for several cellular processes^1^,^12^,^13^. Many studies over the past two decades showed that deletions, variations or mutations within ZnT-1-10 cause severe abnormalities and diseases^12^,^14^. ZnT-10 (SLC30A10), for example, is a manganese transporter that is mainly expressed in liver and brain cells and was shown to be located in the cell membrane, in endosomes and at the Golgi apparatus^15^,^16^,^17^,^18^,^19^,^20^. The ZnT-10 missense mutation L349P in its CTD was shown to be related to high levels of manganese in whole-blood (hypermanganesemia), hepatomegaly and dystonia^21^. Unfortunately, to date there is no biochemical analysis in general and structural characterization in particular of ZnT proteins, which complicates the analysis of their molecular mechanism. However, recently it was shown that the bacterial ZnT homolog MamM can serve as an alternative to study molecular mechanisms of ZnT members^22^.

MamM is a CDF protein exclusively found in magnetotactic bacteria (MTB)^5^,^23^. MTB are a group of Gram negative microorganisms that have the ability to orient themselves along the geomagnetic field, which facilitates their search for microoxic habitats within aquatic sediments^24^. In the model strain *Magnetospirillum gryphiswaldense* MSR-1 and related MTB, this behavior is based on the formation of magnetosomes, unique sub-cellular organelles that are composed of nanometer-sized crystals of the magnetic mineral magnetite (Fe_3_O_4_) surrounded by a lipid bilayer membrane^25^. It was recently proposed that the magnetosome membrane-associated protein MamM is a CDF protein that acts as a magnetosome-directed iron transporter, as its deletion or single point mutations within its CTD abolished magnetite biomineralization. Additionally, mutations within the conserved TMD metal transport site and within the CTD caused alterations in magnetite crystal sizes and morphologies^5^,^22^,^23^. Recent structural and functional studies of this protein confirmed that MamM shares the common CTD structure, dimer fold and conformational changes of other CDFs^5^,^23^.

In order to gain a better understanding of the molecular mechanism of CDF proteins and the specific role of their cytoplasmic domain, it is crucial to investigate the relationship between their structure and function. In this study we applied a combination of computational, biophysical and molecular approaches to analyze the role of CTDs in CDF transporters using MamM as a model system. To this end, we introduced a mutation homologous to the ZnT-10 disease-related L349P mutation into MamM. Based on structural models and molecular dynamics simulations we show that the L349P mutation in ZnT-10 and the corresponding M250P mutation in MamM will cause significant structural changes. Size-exclusion chromatography with multi-angle light scattering, small-angle X-ray scattering, circular dichroism measurements and 1D ^1^H-NMR of the purified MamM M250P CTD revealed a complete loss of structure. Additionally, we show that the MamM M250P mutation causes reduced protein stability but maintains residual activity *in vivo*. Thus, our data provides evidence for the importance of the CTD fold for MamM function and supports the proposed regulatory mechanism for CTD-containing CDFs. Furthermore, based on our results we hypothesize how L349P exerts its influence on ZnT-10’s structure and function.

## Results

### Structural modeling of ZnT-10 CTD

**Figure 1:**
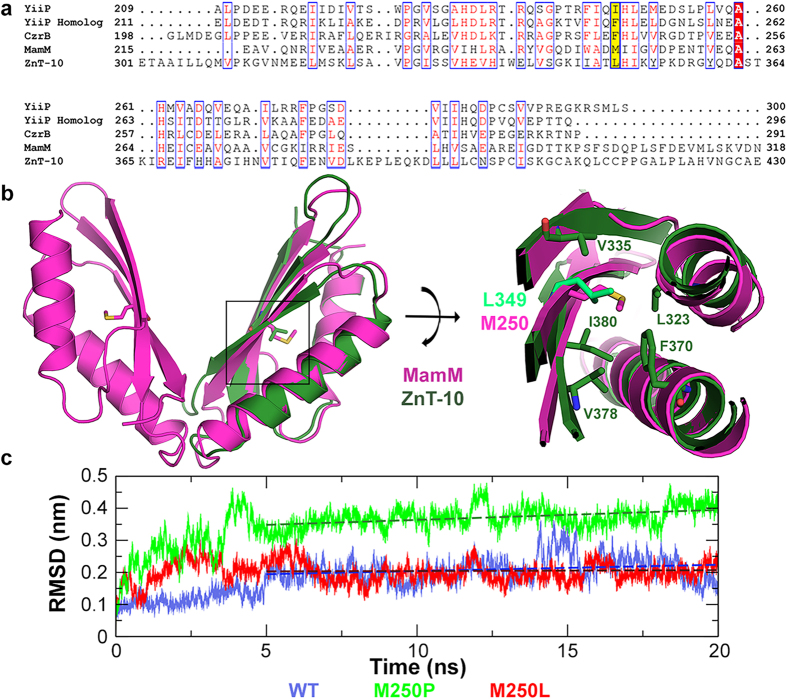
ZnT-10 CTD model, MamM mutation site selection and *in silico* analysis of mutated MamM CTD. (**a**) MSA of ZnT-10 CTD against different CDFs’ CTDs with known structures: YiiP (*Escherichia coli*), YiiP homolog (*Shewanella oneidensis* MR-1), CzrB (*Thermus thermophiles* HB8) and MamM (*Magnetospirillum gryphiswaldense* MSR-1). L349 from ZnT-10 and homologous amino acids in all other organisms are colored in yellow. (**b**) Max Planck Bioinformatics ToolKit Modeller structural model of ZnT-10 CTD (green) overlapped on the MamM CTD dimer structure (3W5Y, pink). Left: ZnT-10 model shows the characteristic fold of CDFs’ CTDs. The ZnT-10 L349 homologous amino acid in MamM is M250; both are found in the middle of the central β-strand facing towards a hydrophobic cavity between the α-helices and the β-sheet. Right: magnification of the rotated black box; M250 in MamM, L349 in ZnT-10 and several of the surrounding hydrophobic residues in ZnT-10 are presented. (**c**) Represented backbone atoms’ RMSDs as a function of time for representative trajectories of MamM CTD WT (purple), M250L (red) and M250P (green). Whereas all simulated systems undergo an initial structural rearrangement stage, WT and M250L show lower RMSDs than M250P, compared to the initial structure. Broken lines show RMSD trends of the last 15 ns of simulations of each system, R2 were calculated to be 0.04982 (WT), 0.00163 (M250L) and 0.19353 (M250P).

To analyze the role of the CTD for overall CDF function we aimed to study the disease-causing ZnT-10 L349P mutation by using MamM as a homologous system. Therefore, multiple sequence alignment (MSA) analyses were performed using different combinations of CDF protein sequences (full-length or only CTD; example in Fig. 1a). Since the CTDs of CDF transporters are only poorly conserved at the sequence level, inconclusive results were obtained from several alignments. However, a large number of the MSAs showed that L349 in ZnT-10 is homologous to M250 in MamM.

Despite their high degree of sequence divergence, all CDF CTDs share a similar metallochaperone-like fold. Therefore, homology models of ZnT-10 CTD were built and overlapped with known MamM CTD structures to ensure the position of ZnT-10 L349. The ZnT-10 CTD structure was predicted using the SWISS-MODEL Automatic Modelling Mode^26^,^27^,^28^,^29^ and Max Planck Bioinformatics ToolKit Modeller (Modeller)^30^ based on known CDF structures^5^,^6^,^7^,^8^,^9^ (RMSD between models: 1.18 Å, 236 atoms involved). ZnT-10 CTD models are very similar and show the characteristic fold of CDF CTDs, containing two α-helices and a three-stranded β-sheet. Leucine 349 is located in the middle of the central β strand, and faces towards a hydrophobic cavity between the β-sheet and the α-helices (Fig. 1b).

Fitting MamM CTD monomer structures (PDB codes: 3W5X and 3W5Y^5^; the latter contains a dimer) onto the ZnT-10 CTD model gives RMSD values of 1.17 Å, 1.30 Å and 1.38 Å (276, 272 and 280 atoms involved, respectively), which reflect their high similarity (for simplicity, only Modeller-predicted structure RMSD scores are presented). M250 of MamM is found at a similar position in the center of the β-sheet as L349 in ZnT-10 (Fig. 1b) and both residues have similar properties.

To ensure that the MamM CTD can serve as a reliable model that will allow us to gain better knowledge of the disease-causing mutation in ZnT-10, we performed *in vitro* studies of MamM CTD M250L, as a representative of wild-type (WT) ZnT-10. We overexpressed and purified MamM CTD M250L using similar protocols to those of MamM CTD WT^31^ (see Supplementary Fig. S1 and *Methods*). Crystallization trials of 6xHis-tagged MamM CTD M250L produced a crystal that diffracted to 1.79 Å resolution (see Supplementary Table S1). The crystal structure shows high similarity to MamM CTD WT protein (PDB code: 3W5X^5^) with an RMSD score of 0.21 Å (316 atoms involved, for further details see Supplementary Fig. S2), indicating that the leucine maintains the structure of the WT MamM CTD.

### *In silico* M250P substitution destabilizes MamM CTD

In order to estimate the effect of the WT-representative M250L and the disruptive M250P mutations on the MamM CTD, we calculated their stability using FoldX force field. This is an empirical force field that has been designed for the calculation of free energies of macromolecules based on their 3D structures^32^. The free energy difference following the M250L substitution was found to be 1.6 Kcal mol^−1^. On the other hand, the free-energy difference following the M250P mutation was found to be 15.8 Kcal mol^−1^. Since proline is the only imino acid found in proteins, it is likely that the origin of the effect of the M250P mutation is mainly due to the accompanying increase in rigidity and/or loss of inter-strand H-bonds. The structural nature of the mutation and the fact that the destabilization energy due to the M250P mutant is uncommonly high^33^ together suggest that the mutation likely distorts the CTD fold or prevents the protein from folding.

To understand the effect of the proline substitution on a folded dimeric MamM CTD structure at atomic resolution, we performed molecular dynamics (MD) simulations. As a control, we also assessed if the substitution M250L will result in destabilization of the MamM CTD. We simulated MamM CTD WT, M250L and M250P in the presence of explicit water molecules and in all cases we observed an initial stage of structural rearrangement followed by a plateau of RMSD values at the productive stages (Fig. 1c). Calculated running averages at the productive stage indicate that WT and M250L systems stabilized after 3 to 5 ns of simulations. On the other hand, the running average calculated from the RMSD of the productive stage of the M250P simulations increased over time, indicating that this mutation destabilizes the protein.

### MamM CTD WT and M250P have different oligomerization states

The results of the 3D-structure stability calculations and the MD simulations suggested significant structural changes in the MamM CTD structure as a result of the M250P substitution. Therefore, we investigated the structure of the mutated protein *in vitro* and used MamM CTD WT and M250L as controls (for detailed expression and purification protocols of all proteins, see *Methods* and Supplementary Fig. S1). Previous studies have shown that MamM CTD WT forms a stable homodimer in solution^5^. However, size-exclusion chromatography (SEC) showed that M250P elutes at a different volume compared to WT and M250L (see Supplementary Fig. S1). Since these results are indicative of a different shape or oligomerization state, we examined whether the dimerization state of the protein is preserved. Size-exclusion chromatography with multi-angle light scattering (SEC-MALS) of M250P showed that the protein has a molecular weight of 12 ± 3 kDa – the molecular weight of a monomer – when eluting from SEC (see Supplementary Fig. S3), suggesting that the protein elutes as a disordered monomer.

### MamM CTD M250P is unstructured

**Figure 2:**
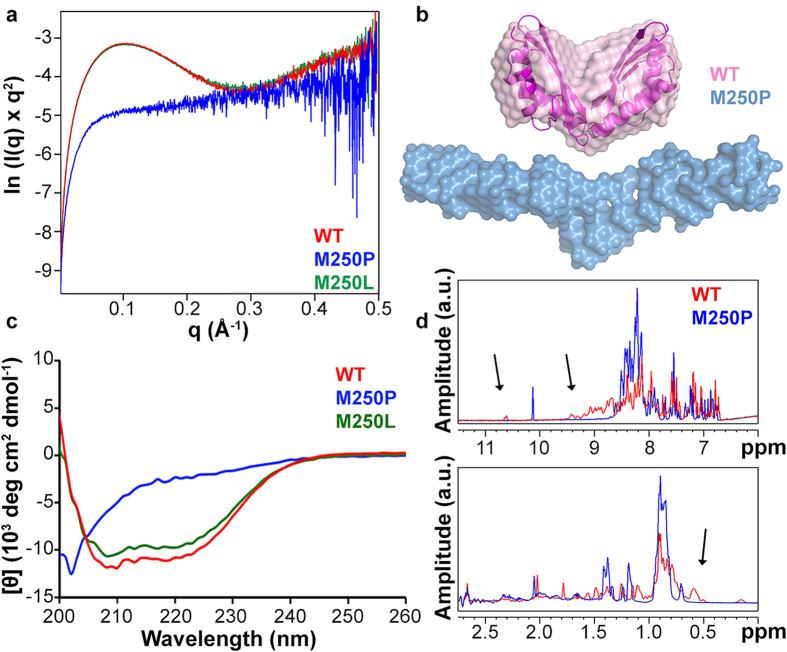
MamM CTD is unfolded in solution. (**a**) SAXS ln-Kratky plots of MamM CTD WT (red), M250L (green) and M250P (blue) show that WT and M250L proteins fold into the same globular-like shape, while M250P Kratky plot has the characteristic shape for unfolded proteins (ESRF BM29). (**b**) SAXS envelope of MamM CTD WT in red with MamM CTD dimer structure fitted inside, taken from Zeytuni *et al*. 2014a, and of MamM CTD M250P in blue. Mutated protein shows a more elongated envelope that cannot fit the MamM CTD native structure. (**c**) CD curves of MamM CTD WT (red), M250L (green) and M250P (blue) show significant differences between the secondary structures of WT/M250L (similar to one another) and M250P proteins. MamM CTD M250P has no clear secondary structures, which suggests it is unfolded even in the secondary structure level. (**d**) NMR shows loss of structure for the M250P mutant. ^1^H-NMR spectrum of WT (red) and M250P mutant (blue) MamM CTD at 80 μM in 10 mM d_11_-Tris buffer pH 7.1, 150 mM NaCl, 0.5 mM β-mercaptoethanol and 7% D_2_O. Resonances in the 8.5–9.5 ppm region, the downfield indole proton at 10.5 ppm (upper panel) and the upfield methyl signals in the 0–0.6 ppm region (lower panel, shown at 180° phasing for clarity) are marked with arrows.

Our *in silico* studies indicated a destabilization of the MamM CTD structure by M250P, which was supported by the change in its oligomerization state. To further examine the effects of the MamM CTD M250P exchange, we used several biophysical techniques. Small-angle X-ray scattering (SAXS) detects low-resolution (nm-scale) structures of proteins in solution and enables the modeling of molecular envelopes. First, we analyzed MamM CTD WT and M250L, which both yielded SAXS Kratky plots similar to a folded lysozyme plot, which was previously used as a globular-protein control^34^. In contrast, MamM CTD M250P displays similarity to a Kratky plot of denatured lysozyme (8 M urea, 90 °C; Fig. 2a)^34^. In order to assess if M250P is truly unfolded and to what extent, this experiment was complemented with SAXS measurements of MamM CTD WT protein with different urea concentrations. It can be assumed that most proteins are unfolded in high urea concentrations (~6 M), while in lower urea concentrations proteins are partially unfolded or unstable^35^. Kratky plots of the SAXS data show a peak that decays as more urea is added, resembling a less globular protein when the urea concentration is increased. More importantly, Kratky plots of MamM CTD M250P (without urea) resemble those of MamM CTD WT with 6 M or 8 M urea (see Supplementary Fig. S4), suggesting that M250P is unfolded in solution. Additionally, while the known crystallographic structure of MamM CTD dimer fits to the WT protein SAXS envelope^5^, the M250P SAXS envelope is elongated and may contain one unfolded monomer (Fig. 2b), as expected from the SEC-MALS results.

The unfolded state of MamM CTD M250P that was detected in the SAXS measurements can be caused by a loop breakage or secondary structure loss. To investigate if the secondary structure changed due to the mutation, circular dichroism (CD) was performed. MamM CTD WT and M250L spectra are very similar and show high content of α-helices, in agreement with their crystal structures. In contrast, the M250P spectrum is significantly different from the WT spectrum and is similar to a typical spectrum of random-coil proteins (Fig. 2c), indicating loss of secondary structures.

To further investigate the structural properties of MamM CTD and its M250P mutant we utilized 1D ^1^H-NMR. Structured proteins characteristically exhibit a widely dispersed chemical shift map due to field effects resulting from the tertiary fold. Conversely, disordered proteins typically exhibit low dispersion as most resonance frequencies cluster around their random coil values^36^,^37^. Since the ^1^H nucleus is one of the most sensitive to such structure-induced effects, the 1D ^1^H-NMR spectrum of proteins is a convenient (as it does not need isotopic labeling) and reliable reporter on the degree of protein disorder. We compared the spectra of MamM CTD and its M250P mutant at 80 μM under native conditions. As demonstrated in Fig. 2d, several features of these spectra indicate that the MamM WT CTD is well structured, whereas the M250P exchange induces an overall loss of structure. In particular, the 8.5–9.5 ppm region typical of structured proteins is populated in the WT protein but disappears in the mutant spectrum, and a shift of outlying peaks (e.g. the Trp indole NH proton and methyl signals) from average values is observed for the WT protein alone. These findings clearly establish the M250P mutant as a disordered protein in contrast to the globular MamM WT CTD.

### MamM M250P function significantly decreases *in vivo*

**Figure 3:**
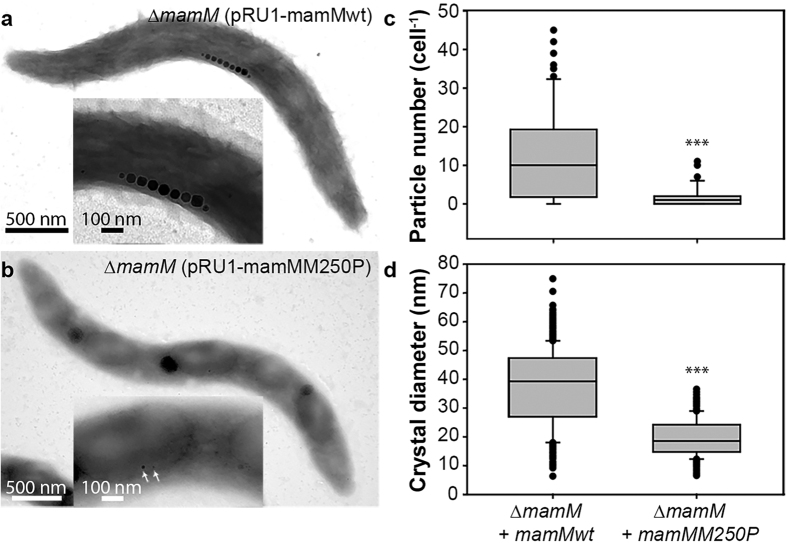
*In vivo* effects of MamM M250P on crystal number per cell, crystal size and crystal shape. (**a**) Representative TEM image of a cell expressing WT *mamM*. Inset: magnification of midcell. (**b**) Representative TEM image of a cell expressing *mamMM250P*. Inset: magnification of midcell with arrows pointing to small magnetosome particles. (**c**) Box plot showing the distribution of crystal numbers per cell from *ΔmamM* strains expressing WT *mamM* (n = 86) and *mamMM250P* (n = 76). Statistical significance of alterations from the strain expressing wild-type *mamM* was tested using the Mann-Whitney test (***P < 0.001). (**d**) Box plot showing the magnetite crystal size distribution of *ΔmamM* strains expressing WT *mamM* (n = 512) and *mamMM250P* (n = 230). Statistical significance of alterations from the strain expressing WT *mamM* was tested using the Mann-Whitney test (***P < 0.001).

To test the effects of the M250P exchange in MamM *in vivo*, replicative pRU1 plasmids with *mamMwt* or *mamMM250P* alleles were transferred to the non-magnetic *mamM* deletion mutant of *M*. *gryphiswaldense* by conjugation. First, we analyzed the ability of the different alleles to restore magnetite biomineralization and cellular magnetic response (C_mag_) in *∆mamM* by a light scattering assay^38^. While trans-complementation of *∆mamM* with *mamMwt* restored C_mag_ to 0.69 ± 0.05, cells expressing *mamMM250P* showed a strongly decreased C_mag_ of 0.04 ± 0.02, indicating a severe impairment of magnetite biomineralization. Next, we analyzed the trans-complemented strains by transmission electron microscopy. As described earlier^5^, trans-complementation of *∆mamM* with pRU1-*mamMwt* restored magnetite biomineralization and resulted in magnetosomes with WT-like magnetite crystal diameters (38 ± 13 nm) but 50% reduced magnetite particle numbers per cell (13 ± 12 cell^−1^), which is partially caused by a substantial number of cells within the population that remained non-magnetic (16.3%). In contrast, cells expressing *mamMM250P* showed significantly decreased magnetite particle diameters (20 ± 7 nm) and numbers (2 ± 2 cell^−1^; Fig. 3), whereas an increased fraction of cells lacked magnetosomes (42%).

Since our *in vitro* experiments indicated that MamM M250P may be unstable we also tested for expression of MamM in the different *M*. *gryphiswaldense* mutant strains by western blot. As described previously^23^, deletion of *mamM* caused degradation of MamB, another CDF protein found in the magnetosome membrane that is stabilized by MamM, which thus could not be detected in whole cell extracts of ∆*mamM*. Trans-complementation of the ∆*mamM* strain with the *mamMwt* alleles restored MamM as well as MamB to WT-like levels. However, in whole cell extracts of *mamMM250P* trans-complemented cells, the amount of both MamM (based on signal intensity by 50–60%) and MamB (based on signal intensity by 40–50%) was significantly reduced (see Supplementary Fig. S5).

## Discussion

**Figure 4:**
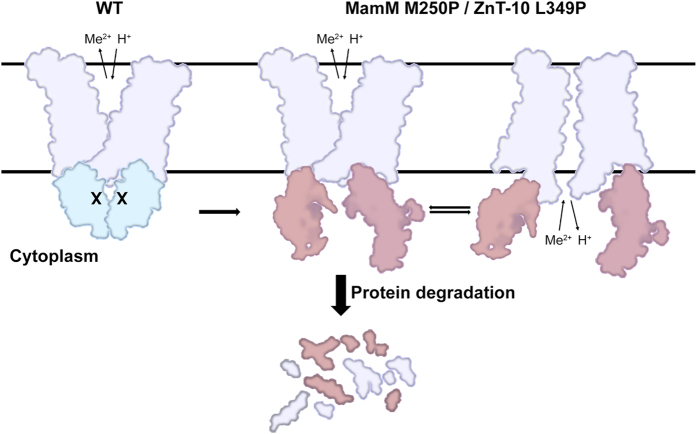
Schematic model of the suggested mechanism of MamM M250P/ZnT-10 L349P. Both WT CDF proteins have the characteristic CDF fold but while the transmembrane domain can be folded into the membrane and create a dimer when mutating the specific position to proline, the cytoplasmic domain cannot fold properly and is at least partially unfolded. This can lead to the recruitment of proteases for misfolded protein degradation; meanwhile the protein functions abnormally, enabling limited transport of metal cations from the cytoplasm.

The C-terminal domain of CDF proteins is crucial for their proper function, yet knowledge of the structure-function relationship of this specific domain is limited. Here, we studied the MamM M250P mutation that is homologous to the disease-causing ZnT-10 L349P mutation, as shown by the high similarity between the tertiary structure of ZnT-10 CTD models and the MamM CTD (RMSD of 1.17–1.38 Å). In all models, L349 of ZnT-10 and M250 of MamM were located at similar positions within the middle of the central β-strand in the β-sheet. This location in the CTD is prone to severe structural changes upon amino acid substitution to proline, as shown by our MD simulations, SAXS, CD and 1D-^1^H-NMR analyses of MamM CTD, and influences the protein function, as reflected in our study and in the genetic study that associated this mutation with hypermanganesemia^21^. We hypothesized that the structural disruption is a specific result of the proline substitution, which restricts phi and psi angles of the α-carbon and, therefore, is incompatible with the structure of stranded β-sheets^39^,^40^,^41^. Additionally, compared to all other amino acids, proline lacks one amide proton that may be required to form a hydrogen bond that stabilizes the β-sheet configuration^41^. In agreement with these constraints, proline is rarely found in β-sheets^42^,^43^ and was shown to impact the overall folding and function of many proteins such as Tau^44^, CFTR^45^,^46^, and EscN^47^. Accordingly, all the *in vitro* experiments with MamM CTD M250P showed that the proline substitution causes severe structural changes while the M250L mutant preserved the WT CTD structure. As a consequence of these structural changes, we also determined using SEC-MALS that MamM CTD M250P does not form dimers but stays in a monomeric form *in vitro*. While the combination of SAXS, CD and ^1^H-NMR results showed that MamM CTD M250P has no secondary structure, our *in silico* approaches predicted that the protein is not completely unfolded but rather cannot fold properly and, therefore, is less stable than the WT protein. These predictions were confirmed by our *in vivo* results since a *mamMM250P* allele partially restored magnetite biomineralization in the non-magnetic Δ*mamM* strain and MamM M250P showed a lower expression level than the WT control. Thus, we propose a mechanism in which the N-terminal transmembrane domain of the mutated protein – a physically separated domain that is thought to be stable by itself, since some CDF proteins lack a CTD^11^ – starts to fold into the membrane, still creating a dimer, but the cytoplasmic domain fails to fold correctly. Although some secondary structures can be formed *in vivo* because of TMD constraints, the partially unfolded state is unstable so it can only moderately function until proteases are recruited for protein degradation (Fig. 4).

The results of this study support the suggestion that the CTDs of CDF proteins have an important role in the regulation of metal transport through the membrane. Several studies have already shown that mutations within the MamM CTD affect the function of the whole protein^5^,^22^,^23^. Partial deletions of the MamM CTD had no effects as long as the folded domain of the MamM CTD (residues 215–293) was preserved^23^. However, MamM was inactive when the last 60 residues, which include the last α-helix and the last β-strand, or more, were deleted^23^. This, together with the M250P mutation – which creates an artificial state where one domain is unfolded – supports the importance of the whole folded domain for proper function. Nevertheless, since in the *mamMM250P* strain a minimal function was observed, as opposed to no function in the deletion of the whole CTD, our results imply that the CTD native structure is not the only functional determinant. Previous studies identified specific amino acids within the CTD that take part in the transport regulation and the dimerization stability of the CTD^22^,^23^. For example, disruption of both putative metal-binding sites in the MamM CTD (D249A-H264A) resulted in a significantly lower magnetic response and number of magnetic particles^5^. V260 is found at the dimerization interface of MamM CTDs and substitutions of this residue (V260D, V260R and V260P) yielded the only mutated forms of MamM full-CTD that showed no magnetite formation *in vivo*. All V260 substitutions are thought to alter the dimerization stability and the ability of MamM to induce proper conformational changes of the TMD to allow cation transport^5^,^22^. We speculate that in contrast to the V260 substitutions, the unstructured cytoplasmic domain does not interrupt the TMD structure and does not physically limit the TMD conformational changes. This, together with the presence of the putative binding-site residues in the *mamMM250P* strain, allows for some ion transport through the TMD. Interestingly, in contrast to MamM M250P, all previously-tested point mutations that were tested on MamM (single- or double-mutations) showed WT-like expression levels^5^,^22^. Thus, MamM M250P is the first amino acid exchange applied that showed this phenotype and, together with all the other mutants that were studied, supports the importance of the CTD in the overall protein folding, stability and function.

In light of the MamM results and the current knowledge about ZnT-10 function, we propose a mechanism for how a single nucleotide polymorphism in ZnT-10, T1046C (L349P), can cause high levels of whole-blood manganese (hypermanganesemia), hepatomegaly and dystonia. ZnT-10 is found in cells of the liver and the epithelium of bile ducts, where it localizes to the plasma membrane that faces the lumen of the bile ducts^15^ and most likely has a role in transporting manganese into the bile. Considering the conservation between MamM CTD M250L structure and the MamM CTD structure (see Supplementary Fig. S2), and the conservation between MamM CTD structures and ZnT-10 models (Fig. 1b), we suggest that the ZnT-10 L349P amino acid exchange – similarly to the M250P exchange in MamM – destabilizes ZnT-10 so that the CTD cannot fold properly (Fig. 4). As with MamM M250P, a substantial fraction of misfolded ZnT-10 L349P proteins might be degraded, which in turn would cause manganese accumulation in cells. While we assume that the mutated protein retains a residual manganese transport activity that prevents cell death, cellular manganese accumulation would cause increased manganese levels in the blood due to decreased manganese influx into the cells. Therefore, patients with mutations in ZnT-10 have hepatic dysfunction and increased manganese levels in whole-blood^15^,^21^. Since manganese can cross the blood-brain barrier, elevated blood manganese levels also cause its accumulation in basal ganglia neurons^15^,^18^,^48^ resulting in parkinsonism-like syndromes, as was demonstrated for L349P and other mutations in ZnT-10^15^,^21^,^49^.

To conclude, our study shows how a single mutation in a CDF protein’s CTD can cause its structural loss and leads to dysfunction of the whole protein. Furthermore, our results emphasize the importance of CDF cytoplasmic domains as regulatory domains that are crucial for the proper transport ability of CDF proteins.

## Methods

### Least-squares overlaps and multiple sequence alignment

ZnT-10 CTD models, MamM CTD WT (PDB codes: 3W5X, 3W5Y) and M250L structures were overlapped and root-mean-square deviations (RMSDs) were calculated using the iterative magic fit and fragment alternate fits of Swiss-PdbViewer 4.1.0 (best fit was selected)^50^. ZnT-10 CTD models, M250L CTD structure and all overlapped structures’ figures were prepared using PyMOL^51^. MSA was performed using the ClustalW2 server^52^,^53^.

### Molecular dynamics

In this study, three systems were simulated: WT, M250P and M250L MamM CTD in a water environment. The atomic coordinates of MamM CTD WT were taken from the crystal structure of the dimer MamM CTD (PDB code: 3W5X^5^). Atomic coordinates of MamM CTD M250P and M250L were achieved by *in silico* mutation of MamM CTD WT using PyMOL version 1.7r4^51^. The protonation state of each protein was assigned at neutral pH (pH = 7) using the program PDB2PQR version 1.9.0^54^,^55^. All molecular dynamics (MD) simulations were performed using the GPU implementation of pmemd^56^ in the AMBER package version 14^57^, in conjunction with the ff14SB all-atom force field^58^. Each protein was first put into a box, of which the minimal distance from the protein to the box wall was 1.4 nm, and then solvated using TIP3P water molecules^59^. No counter ions were used because all simulated systems are neutral. Each system was then subjected to 10,000 steps of energy minimization. Simulated systems were then heated to 298 K with the protein harmonically constrained for 0.1 ns under the NVT conditions, followed by the 0.4 ns MD simulations with harmonic constraints using the NVT ensemble. MamM WT and mutants were simulated twice independently, each system starting with a different distribution of initial velocities.

For all simulations, a time-step of 2 fs was used together with periodic boundary conditions. The non-bonded cutoff distance was 0.9 nm, and the particle mesh Ewald (PME) algorithm was used for the calculation of electrostatic energy^60^. The SHAKE algorithm^61^ was used to constrain the bond lengths to all hydrogen atoms and the internal geometry of all the water molecules. The system was maintained at 298 K using a Langevin thermostat with a collision coefficient of 1 ps^−1^. The reference pressure was set equal to 1 Bar using the Berendsen barostat^62^ with the pressure-coupling constant of 2 ps.

*MD analyses*. Analyses were performed with ptraj^63^ in AmberTools version 15^57^. Visualizations and image rendering were performed using PyMOL software^51^. For RMSD analyses, the positional deviations of backbone heavy-atoms with respect to the initial structure were calculated every 50 ps (after performing a least-squares fit to their initial structure).

### Cloning, site-directed mutagenesis and protein expression

MamM CTD (215–318 a.a.) from *Magnetospirillum gryphiswaldense* MSR-1 was cloned and expressed as described previously^31^. MamM CTD M250P and M250L mutations were applied to the pET28a-MamM-CTD-MSR1 vector using the QuickChange site-directed mutagenesis method (Startagene, CA, US). Primers containing single mutation sites (Hylabs, Israel) were designed and used for PCR amplifications. MamM CTD M250L was expressed as described previously for MamM CTD WT^31^. The plasmid pET28a-MamM-CTD-M250P was transformed into *E*. *coli* BL21(DE3) using the heat shock method. Cells were grown in LB broth medium containing kanamycin (50 μg mL^−1^) for 16 h at 310 K, then transformed to a TY-based auto-induction medium, containing 0.5% glycerol, 0.05% glucose, 0.2% α-lactose and 50 μg mL^−1^ kanamycin, at a 1:32 ratio. After 6 h at 310 K, the temperature was reduced to 300 K for 18 h. Cells were harvested via centrifugation at 7438 × *g* for 10 min in 277 K.

### Protein purification

MamM CTD WT and M250L were purified as described previously for WT^31^, with the modification of the Triton-X 100 concentration in buffer A to 0.01% (volume percentage). For crystallization of MamM CTD M250L, a fraction of 6xHis-tagged protein was not cleaved after the Ni-NTA affinity purification step (the same protocol was used, but without thrombin). MamM CTD M250P-expressing cells were suspended in Buffer A (50 mM Tris-HCl pH = 8, 200 mM NaCl, 10 mM imidazole, 5 mM β-mercaptoethanol, 5% glycerol, 0.045% Triton X-100 and 0.03% TWEEN 20) at a weight ratio of 1:2, with DNaseI (10 μg mL^−1^) and a protease inhibitor cocktail (containing phenylmethylsulfonyl fluoride (PMSF), 100 μM; leupeptin, 1.2 μg mL^−1^; and pepstatin A, 1 μM) for 20 min at 277 K. Suspended cells were then disrupted by three cycles of French press pressure cell (Thermo Scientific, NC, US) at 207 MPa and centrifuged at 45,000 RPM (60 Ti fixed angle rotor, Beckman Coulter, CA, US) for 45 min at 277 K. Supernatant fraction was applied to a home-made gravity HIS-Select Cobalt Affinity Gel (5 mL bead volume; H8162, Sigma-Aldrich, Israel) in an Econo-Column (Bio-Rad, CA, US) chromatography column that was pre-equilibrated with buffer A. Protein was washed with two washing buffers for further purification: Buffer B (20 mM Tris-HCl pH = 8, 500 mM NaCl, 10 mM imidazole, 5 mM β-mercaptoethanol and 5% glycerol) and Buffer C (20 mM Tris-HCl pH = 8, 150 mM NaCl, 10 mM imidazole, 5 mM β-mercaptoethanol and 5% glycerol) and eluted using Buffer D (20 mM Tris-HCl pH = 8, 150 mM NaCl, 500 mM imidazole, 5 mM β-mercaptoethanol and 5% glycerol). In order to cleave the 6xHis-tag, bovine thrombin (1 U mL^−1^; t4648-10 KU, Sigma-Aldrich) was added to the eluted protein and the mixture was dialyzed for 16 h at 277 K against Buffer E (10 mM Tris-HCl pH = 8, 150 mM NaCl and 5 mM β-mercaptoethanol). Protein was then concentrated to a volume of 4 mL using a Vivaspin-4 (3000 Da Mw cutoff; Sartorius Stedim Biotech, Germany) and applied onto a HiLoad 26/60 Superdex75 size-exclusion column (GE Healthcare, UK), pre-equilibrated with Buffer E. Selected peaks of pure protein were collected and concentrated using a Vivaspin-4 to a final concentration of 18 mg mL^−1^, flash-frozen in liquid nitrogen and stored at 193 K for further use. Protein concentration was determined by measuring protein absorption at 280 nm, protein purity was analyzed by SDS-polyacrylamide (20%) gel electrophoresis (PAGE) and protein identification was confirmed by western blot using HRP-conjugated anti-6xHis-tag antibody (ab1187, Abcam, UK) after affinity column and tandem mass spectroscopy.

### Crystallization and structure determination

Purified 6xHis-MamM CTD M250L at 5 mg mL^−1^ concentration in Buffer E was crystallized using the vapor diffusion method at 293 K (0.3 μL protein with 0.3 μL reservoir solution: 0.2 M (NH_4_)_2_SO_4_, 0.1 M BIS-TRIS pH = 5.0 and 25% PEG 3350). Crystals were harvested immediately after the addition of a cryo-protection solution (containing 1 μL reservoir solution and 4 μL 50% PEG 3350) into the drop and flash-frozen in liquid nitrogen. Data collection was performed on a single-crystal at a temperature of 100 K at the P13 EMBL beamline in the Deutsches Elektronen-Synchrotron (DESY), Hamburg, Germany. Data reduction and scaling were performed with XDS^64^ and phases were obtained by the molecular replacement method with the MamM CTD WT structure (PDB code: 3W5X^5^) as a template using the Phaser molecular-replacement^65^ in the CCP4 package^66^. The structure was refined by REFMAC5^67^ and Coot^68^ and the final model was refined and built by the PDB_REDO server^69^. Rfree calculation used 5% of the data. Further details in Supplementary Table S1.

### Size-exclusion chromatography with multi-angle light scattering

SEC-MALS measurements for MamM CTD M250P were performed in a SEC-MALS system consisting of an AKTA Explorer (GE), MiniDawn TREOS and OPTILAB T-reX (Wyatt Technology, CA, US). MamM CTD M250P was pre-diluted to a final concentration of 4 mg mL^−1^ in Buffer F (containing 10 mM Tris-HCl pH = 8, 50 mM NaCl and 5 mM β-mercaptoethanol). A Superdex75 column 10/300 GL (GE) was pre-equilibrated with Buffer F, following which 200 μL of the protein was injected. Detection was performed using three detectors: refractive index, ultra violet at 280 nm and multi-angle laser-light scattering. Internal calibration was performed with bovine serum albumin, ovalbumin, ribonuclease A and aprotinin.

### Small-angle X-ray scattering

MamM CTD WT, M250L and M250P samples were pre-diluted to final concentrations of 10 mg mL^−1^ (0.42 mM dimer, WT and M250L) and 5 mg mL^−1^ (0.42 mM monomer, M250P) in Buffer E. MamM CTD WT samples were also diluted in Buffer E containing 2, 4, 6 or 8 M of urea to a concentration of 10 mg mL^−1^.

*MamM CTD WT*, *M250L and M250P*. SAXS patterns of MamM CTD WT, M250L and M250P protein solutions were measured at the BM29-bioSAXS beamline at the European Synchrotron Radiation Facility (ESRF) in Grenoble, France. An energy of 12.5 keV corresponding to a wavelength of 0.998 Å^−1^ was selected. The scattering intensity was recorded using a Pilatus 1M detector, in the interval 0.004 < q < 0.5 Å^−1^. Ten frames with 2 s exposure times were recorded for each sample. Measurements were performed in a flow mode where samples were pumped through the capillary at a constant flow rate. The dedicated beamline softwares BsxCuBe and EDNA were used for data collection and processing.

*MamM CTD WT with urea*. SAXS patterns of MamM CTD WT with urea concentrations of 0, 2, 4, 6 and 8 M were measured using a SAXSLAB GANESHA 300-XL. Cu Κα radiation was generated by Genix 3D Cu-source with integrated Monochromator, three pinholes collimation and two-dimensional Pilatus 300K detector. The scattering intensity was recorded in the interval 0.012 < q < 0.7 Å^−1^. The scattering curves were corrected for counting time and sample absorption. No attempt was made to obtain absolute units. The solutions under study were sealed in a thin-walled capillary (quartz) of approximately 1.5 mm diameter and 0.01 mm wall thickness; the temperature was controlled using a Julabo temperature control unit and measurements were performed under vacuum at 277 K.

*SAXS analysis*. The 2D SAXS images were azimuthally averaged to produce one-dimensional profiles of intensity – I vs. q – using the two-dimensional data reduction program SAXSGUI (JJ X-Ray Systems ApS, Denmark). The scattering spectra of the capillary and solvent were also collected and subtracted from the corresponding solution data using the Irena package^70^ for analysis of small-angle scattering data. MamM CTD M250P molecular envelope was created using the DAMMIF and DAMAVER softwares^71^ installed on the BsxCuBe module and visualized using PyMOL^51^. Further analyses and final plot preparations were performed using IGOR Pro (WaveMetrics Inc., OR, US) and the ATSAS suite^72^.

### Circular dichroism

Circular dichroism measurements were performed using a J750 spectropolarimeter (Jasco Inc, ND, US). MamM CTD WT, M250L and M250P were pre-diluted to a final concentration of 0.3 mg mL^−1^ in Buffer G (10 mM Tris-HCl pH = 8, 50 mM NaCl and 0.375 mM β-mercaptoethanol) and measured with a 0.1 cm optical path Suprasil quartz cuvette (Hellma GmbH & Co., Germany). Spectra profiles of the samples were measured at ambient temperature in a wavelength range of 200–260 nm, with bandwidth of 1 nm, scan speed of 5 nm min^−1^ and time constant of 8 s.

### ^1^H-NMR data acquisition

For acquisition of NMR data, MamM CTD samples (WT and M250P) were prepared in 10 mM d_11_-Tris buffer (Cambridge Isotope Laboratories, MA, US) titrated to pH 7.1, 150 mM NaCl, 0.5 mM β-mercaptoethanol and 7% D_2_O. Final protein concentration was 80 μM. ^1^H spectra were acquired on a DRX 700 MHz spectrometer equipped with z-gradients and a cryoprobe at 293 K. Water suppression was achieved using a combination of jump-return^73^ and WATERGate^74^ suppression blocks. Under these conditions spectra were obtained in 10–15 minutes each.

### Bacterial strains, oligonucleotides and plasmids for *in vivo* characterization

Bacterial strains, plasmids and oligonucleotides used in this study are listed in Supplementary Table S2. All strains were cultivated as described previously^5^,^22^.

### Trans-complementation assays

For trans-complementation of *∆mamM*, pRU1-*mamMwt* and pRU1-*mamMM250P* were transferred to *∆mamM* by conjugation^23^. After plasmid transfer, the average magnetic response (C_mag_) of three independent trans-conjugants was assayed^38^. Briefly, using a permanent magnet cells were aligned at different angles relative to the light beam of a UV–vis spectrophotometer (Ultrospec 2100 pro, GE Bioscience, MO, US). The ratio of the resulting maximum and minimum scattering intensities (*C*_mag_) was correlated with the average number of magnetic particles. Imaging of trans-complemented cells by transmission electron microscopy (TEM) was performed as previously described^75^. Briefly, unstained cells were adsorbed on carbon-coated copper grids, air-dried (Plano GmbH, Germany), and analyzed with a Zeiss CEM 902 A transmission electron microscope (Carl Zeiss, Germany) at an accelerating voltage of 80 kV.

Expression of *mamM* and site-directed variants was tested by separation of 10 μg of whole cell protein by SDS-PAGE (12%) and subsequent western blot analysis, as previously described^23^.

## Additional Information

**Accession codes:** MamM CTD M250L atomic coordinates and structure factors have been deposited in the Protein Data Bank (accession number: 5HSP).

**How to cite this article**: Barber-Zucker, S. *et al*. Disease-Homologous Mutation in the Cation Diffusion Facilitator Protein MamM Causes Single-Domain Structural Loss and Signifies Its Importance. *Sci. Rep.* **6**, 31933; doi: 10.1038/srep31933 (2016).

## Supplementary Material

Supplementary Information
