# Supplementary material for: Disease-Homologous Mutation in the Cation Diffusion Facilitator Protein MamM Causes Single-Domain Structural Loss and Signifies Its Importance
Source: Sci Rep. 2016 Aug 23;6:31933. doi: 10.1038/srep31933 (PMC4994047; doi:10.1038/srep31933)
Supplement: Supplementary Information [file srep31933-s1.pdf]

Supplementary Information for:

Disease-Homologous Mutation in the Cation Diffusion Facilitator Protein MamM Causes Single-Domain Structural Loss and Signifies Its Importance

Authors: Shiran Barber-Zucker[1¥], René Uebe[2¥], Geula Davidov[1], Yotam Navon[3], Dror Sherf[1], Jordan H. Chill[4], Itamar Kass[1], Ronit Bitton[3], Dirk Schüler[2] and Raz Zarivach[1]*

Authors' affiliations:

[1] Department of Life Sciences and The National Institute for Biotechnology in the Negev, Ben-Gurion University of the Negev, Beer Sheva, 8410501, Israel.

[2] Department of Microbiology, University of Bayreuth, Bayreuth, 95447, Germany.

[3] Department of Chemical Engineering and Ilse Katz Institute for Nanoscale Science and Technology, Ben-Gurion University of the Negev, Beer Sheva, 8410501, Israel.

[4] Department of Chemistry, Bar-Ilan University, Ramat-Gan, 5290002, Israel.

[¥] These authors contributed equally to this work.

* Correspondence should be addressed to Raz Zarivach, Department of Life Sciences, Ben-Gurion University of the Negev, P.O.B. 653, Beer Sheva 8410501, Israel. Tel: +972-8-6461999, Fax: +972-8-6472970, Email: zarivach@bgu.ac.il

Supplementary Table S1: 6xHis-MamM CTD M250L data collection and refinement statistics.

PDB code	***5HSP***
Data collection	P13 EMBL DESY
Space group	$C222_1$
**Cell dimensions**	
a, b, c (Å)	37.83, 94.91, 53.69
α, β, γ (°)	90, 90, 90
Resolution (Å)	1.79
Rmerge	11.0 (109.2)
I/σI	16.5 (2.2)
$CC_{1/2}$	0.999 (0.667)
Completeness (%)	98.7 (77.9)
Redundancy	12.3
Wavelength (Å)	1.27
No. unique reflections	9313
**Refinement**	
Resolution (Å)	1.79 – 47.46
Rwork/Rfree	20.18/24.31
**No. atoms**	
Protein	619 chain A, 63 chain D
Ligand/ion	10
Water	39
**B-factors**	

Protein	26.37 chain A, 46.69 chain D
Ligand/ion	52.14
Water	44.24
**RMSD**	
Bond lengths (Å)	0.012
Bond angles (°)	1.494
Ramachandran statistics[§]	P: 83 (98.81%), A: 1 (1.19%), O: 0
Missing residues	His-tag 1-18, MamM 292-306, 315-318

Values in parentheses are for the highest resolution shell (1.79 – 1.83 Å).

One crystal was used per dataset.

Data was collected at 100K.

[§] P- Preferred region, A- Allowed region, and O- outliers.

Supplementary Table S2: Bacterial strains, oligonucleotides and plasmids for *in vivo* characterization.

Strains/Oligos/Plasmids	***Important features/Sequences***	***Source/reference***
E. coli		
DH5α	F' Φ80d*lac* ΔM15 Δ(*lacZYA-argF*)U169 *deo*R *rec*A1 *end*A1	Invitrogen
WM3064	*thrB1004 pro thi rpsL hsdS lacZ*ΔM15 RP4-1360 Δ*(araBAD)*567 Δ*dapA1341::[erm pir* (wt)*]*	W. Metcalf
M. gryphiswaldense		
MSR-1 R3/S1	Rif^r, Sm^r spontaneous mutant, wildtype	[1]
Δ*mamM*	R3/S1 but Δ*mamM*	[2]
**Oligonucleotides**		
MamMM250P_fw	GCCGACCCGATTATTGGCGTCGAT	This study
MamMM250P_rev	CCAGATGTCTTGGCCCACATAGCGAG	This study
**Plasmids**		
pRU1	pBBR1MCS-2 with P_{mamAB}	[2]
pRU1-mamMwt	pRU-1 + *mamM*	[2]
pRU1-mamMM250P	pRU-1 + *mamM M250P*	This study

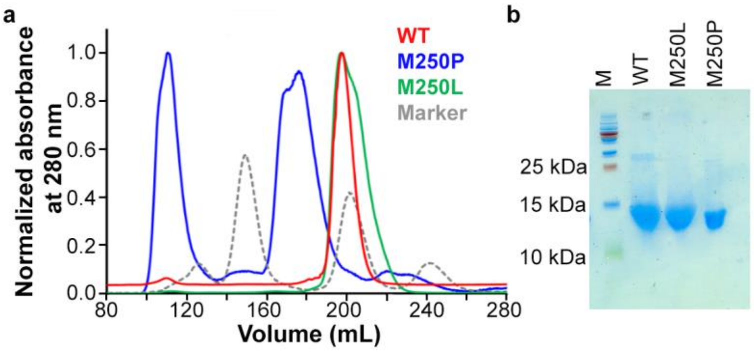

Supplementary Figure S1. Purification of MamM CTD WT, M250P and M250L. (**a**) Size-exclusion chromatograms of MamM CTD WT (red), M250P (blue) and M250L (green) show the same elution volume for WT and M250L and a lower elution volume for M250P, suggesting a different globular fold or oligomerization state resulting from this mutation. Molecular marker in dashed grey line: Conalbumin (75 kDa), Ovalbumin (43 kDa), Ribonuclease A (13.7 kDa) and Aprotinin (6.5 kDa). (**b**) SDS-PAGE of MamM CTD WT, M250L and M250P after size-exclusion shows that all proteins are at the expected size (11.85 kDa monomer) and purified to a satisfactory level for biophysical assays.

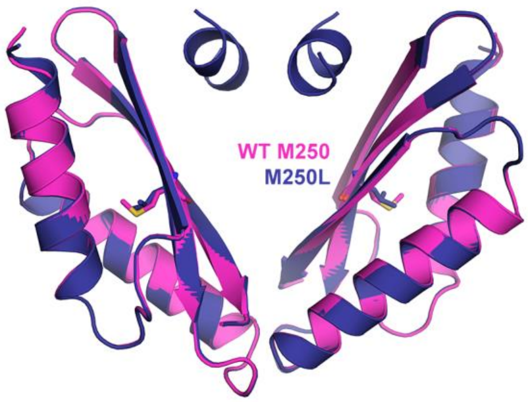

Supplementary Figure S2. The structure of MamM CTD M250L shows high similarity to MamM CTD WT. Overlapping MamM CTD WT (PDB code: 3W5X, pink) and M250L (purple) structures (RMSD 0.21 Å, 316 atoms involved) shows an identical overall structure, which suggests a similar function in the structural stability of the leucine in ZnT-10 and methionine in MamM (both the leucine and the methionine are presented). MamM CTD M250L structure contains a C-terminal α-helix that was not determined before in any MamM CTD structures [3,4]. This new helix (residues 307-314, most likely from a different monomer than the main chain monomer) is now available not due to the leucine mutation – since the structure of the mutated protein's folded domain is essentially the same as that of the WT – but because of the crystallization conditions (different buffers and low concentration of the 6xHis-tagged protein) and crystal forces that stabilized the helix in this conformation. This is supported by a previous study that showed that the MamM 215-293 amino acid construct functioned similarly to MamM 215-318 (full CTD) [3].

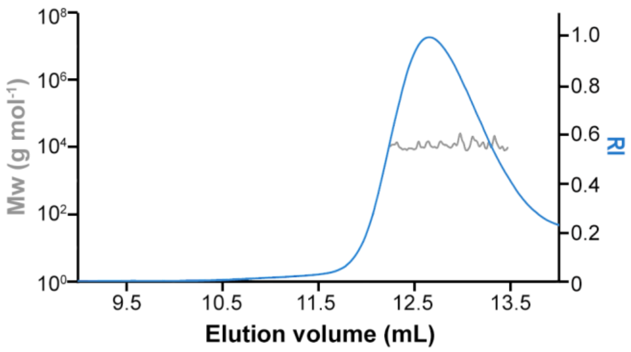

Supplementary Figure S3. Size-exclusion chromatography with multi-angle light scattering of MamM CTD M250P. SEC-MALS results (molecular weight in grey, refractive index in blue) show that MamM CTD M250P has a size of ~12±3 kDa in solution, the size of a MamM monomer.

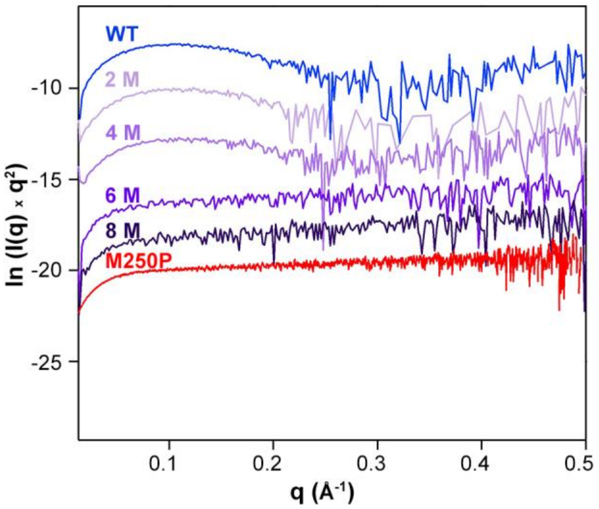

Supplementary Figure S4. SAXS ln-Kratky plot of MamM CTD. Top-to-bottom: WT with no urea, WT with 2 M urea, WT with 4 M urea, WT with 6 M urea, WT with 8 M urea (all home-source) and M250P with no urea (ESRF BM29). The mutant plot is most similar to WT with 6-8 M urea, suggesting that the mutated protein is unstructured in solution.

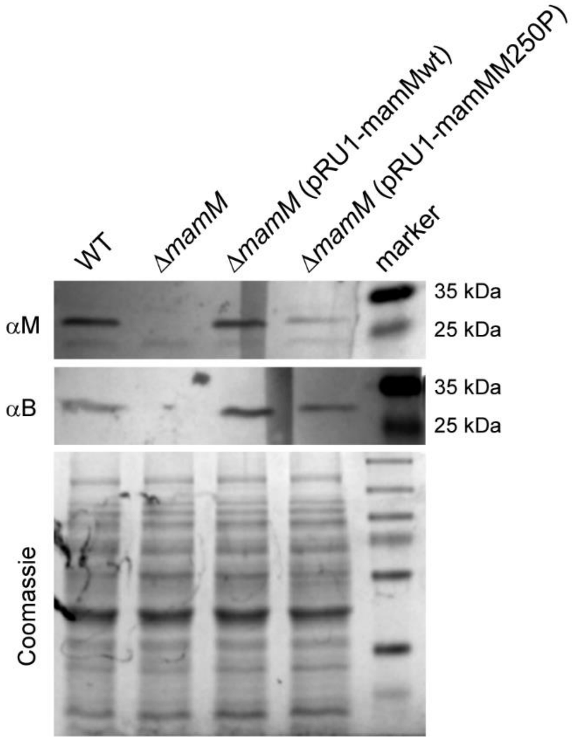

Supplementary Figure S5. SDS-PAGE analysis and immunodetection of MamM and MamB WT and mutant proteins in cell lysates.

Supplementary References:

1. Schultheiss, D., Handrick, R., Jendrossek, D., Hanzlik, M. & Schüler, D. The presumptive magnetosome protein Mms16 is a poly(3-hydroxybutyrate) granule-bound protein (phasin) in *Magnetospirillum gryphiswaldense*. *J. Bacteriol.* **187,** 2416–2425 (2005).

2. Uebe, R. *et al.* The cation diffusion facilitator proteins MamB and MamM of *Magnetospirillum gryphiswaldense* have distinct and complex functions, and are involved in magnetite biomineralization and magnetosome membrane assembly. *Mol. Microbiol.* **82,** 818–835 (2011).

3. Zeytuni, N. *et al.* Cation diffusion facilitators transport initiation and regulation is mediated by cation induced conformational changes of the cytoplasmic domain. *PLoS One* **9,** e92141 (2014).

4. Zeytuni, N. *et al.* Bacterial magnetosome biomineralization - a novel platform to study molecular mechanisms of human CDF-related Type-II diabetes. *PLoS One* **9,** e97154 (2014).
